# Synergistic effects of biochar and microbial inoculants on rice productivity and soil fertility are mediated by a nitrogen-dependent microbial pathway

**DOI:** 10.3389/fpls.2026.1804182

**Published:** 2026-04-14

**Authors:** Qiang Zhang, Mengxuan Gu, Gexing Li, Haoran Jing, Mengying Qi, Hao Li, Mengjie Li, Lingling Ding, Zhi Li, Pengfei Wei, Xiaona Ji, Tao Zhu, Haiying Zhao

**Affiliations:** 1 College of Life Science and Engineering, Henan University of Urban Construction, Henan, Pingdingshan, China; 2 Xinyang Academy of Agricultural Sciences, Xinyang, Henan, China

**Keywords:** biochar, functional prediction, microbial inoculant, microbial-mediated nutrient cycling, nitrogen use efficiency, rice, soil microbiome

## Abstract

Excessive nitrogen (N) fertilizer application threatens soil health and sustainable rice production. We hypothesized that the combined application of biochar and microbial inoculants (B+M) could maintain rice yield under reduced N input through microbiome-mediated mechanisms, and that this effect may depend on an N threshold. To test this hypothesis, pot and field experiments were conducted using the rice cultivar Nanjing 9108 with four treatments: conventional N (CK), and three B+M treatments with 20% N reduction (N80), 40% N reduction (N60), and 0% N reduction (N100). The results indicated that compared to CK, the N100 and N80 treatments increased rice yield by 10.4-11.7% and 4.1-4.9%, and improved Nitrogen Partial Factor Productivity (NPFP) by 7.8-11.7% and 28.6-30.0%, respectively. In contrast, the N60 reduced yield by 3.9-4.6%. In pot experiments, 16S rRNA gene sequencing revealed that N100 and N80 enriched bacterial phyla (e.g., Proteobacteria, Actinobacteria, Chloroflexi, and Bacteroidota) and enhanced microbial functional genes linked to metabolism, genetic information processing, cellular processes, and environmental information processing, thereby increasing soil nutrient availability. Structural equation modeling demonstrated that the soil bacterial community was the fundamental driver of yield enhancement (model fit: χ²/df = 1.13, RMSEA = 0.042, CFI = 0.961). However, under 40% N reduction, the abundance of key phyla declined and microbial functional potential weakened, leading to reduced soil nutrient availability and yield. These findings reveal an N dependent microbial-mediated pathway governing the synergistic effects of biochar and microbial inoculants, identifying 20% N reduction with B+M as an optimal strategy for sustainable rice intensification.

## 1 Introduction

As one of the world’s major food crops, the yield and quality of rice (*Oryza sativa* L.) significantly affect global food security (Heredia et al., 2022). China, being the largest producer and consumer of rice globally, faces a critical challenge in rice production: the widespread overuse of nitrogen (N) fertilizer, which has become a key constraint to enhancing yield and maintaining ecological health (Li et al., 2018; Xu et al., 2023). Therefore, exploring green cultivation practices that reduce N input while enhancing efficiency is of paramount importance. In recent years, the application of biochar and microbial inoculants (B+M) has provided new avenues for sustainable rice production. Biochar is a carbon rich, porous, and solid particulate material produced through the pyrolysis of agricultural biomass under limited or anaerobic conditions. It is characterized by a high degree of aromatization, large specific surface area, strong adsorption capacity, high stability, and abundant surface functional groups (Zhang et al., 2023). Its porous structure and large surface area enable it to adsorb substantial nutrients, while also reducing soil bulk density, improving soil porosity and aggregate stability, thereby enhancing soil physicochemical properties and increasing rice yield (Dong and Lin, 2018; Ruan et al., 2023; Khan et al., 2024). Additionally, biochar improves soil biological properties by increasing microbial population richness, thereby regulating soil nutrient cycling. Chen et al. (2023) reported that combined application of reduced N fertilizer and biochar significantly increased total organic carbon content in paddy soil, elevated the relative abundance of dominant bacterial groups such as Proteobacteria, Actinobacteria, and Gemmatimonadetes, enhanced total soil N, and promoted rice yield. Zhang et al. (2022) showed that biochar input increased the abundance of key bacterial phyla like Acidobacteria and Spirochaetes in paddy soil, and this abundance was positively correlated with yield. Biochar application also enhances the activities of soil enzymes such as dehydrogenase, urease, and catalase, significantly improving soil nutrient availability and rice yield (Yang et al., 2023).

However, in practical application, biochar faces limitations including its limited direct nutrient supply, large volume, and high transportation costs, which restrict its widespread agricultural adoption (Lan et al., 2024). Microbial inoculants contain large numbers of beneficial living microorganisms. When applied to fields, they can colonize and proliferate in the rhizosphere soil, establishing dominant microbial communities conducive to crop growth (Kaya et al., 2023). Nevertheless, a key challenge in using microbial inoculants is the difficulty for the target microorganisms to establish themselves as dominant populations, leading to short effective periods and inconsistent application effects (Fu et al., 2019). The combined application of B+M is proposed to create a synergistic system that mutually compensates for these individual shortcomings. The highly porous structure, large surface area, and favorable surface properties of biochar provide a protected microhabitat for microbial inoculants. This sanctuary shields the introduced strains from predation and competition, facilitating their colonization, survival, and proliferation in the rhizosphere (Bolan et al., 2023; Zhou et al., 2025). Concurrently, the labile carbon fractions and nutrients present in biochar can serve as a transient energy source for these microorganisms, stimulating their metabolic activity and growth. This enhanced microbial activity, in turn, can accelerate the weathering of biochar, potentially releasing some of its bound nutrients, and may improve the biochar’s surface functionality through the production of organic acids and enzymes (Niu et al., 2024). This biochar-microbe feedback loop is believed to be the core mechanism through which the combination enhances soil functions beyond what is achievable by either amendment alone. Zhao et al. (2018) found that compared to the sole application of biochar or microbial inoculant, their combination significantly increased the activities of urease, sucrase, polyphenol oxidase, and catalase in tobacco soil. Furthermore, soluble organic carbon, readily oxidizable organic carbon, and microbial biomass carbon increased by 43.0%, 74.3%, and 99.8%, respectively, compared to chemical fertilizer alone. Wang et al. (2023a) suggested that the synergistic effect of B+M promoted bacterial growth and reproduction, increased bacterial community diversity and the abundance of dominant populations, and improved the structural composition of the bacterial community in rhizosphere soil.

Despite promising findings in upland crops, research on the combined application of B+M in waterlogged paddy ecosystems remains limited. The distinct anaerobic conditions, nutrient cycling processes, and microbial community characteristic of paddy soils may differentially modulate the synergistic mechanisms observed in upland systems. More critically, few studies have systematically examined whether B+M application can mitigate yield losses under N reduction in rice. And it remains unknown whether there exists an N availability threshold below which the beneficial effects of B+M collapse, and whether microbiome-mediated mechanisms underlie such a threshold effect. To address these knowledge gaps, this study integrated agronomic, physiological, and microbiome analyses using the rice cultivar Nanjing 9108. We hypothesized that: (1) combined B+M application maintains rice yield under moderate (20%) N reduction but not under severe (40%) reduction; (2) this yield maintenance is mediated by shifts in soil bacterial community structure and function that enhance nutrient availability; and (3) the synergistic effect depends on an N availability threshold, below which microbial functional redundancy is exceeded, leading to functional decline and reduced productivity. The conceptual framework posits that biochar provides a protective habitat for microbial inoculants, enhancing their colonization and activity, which in turn promotes nutrient cycling and rice physiological performance. However, when N is reduced excessively, microbial communities may shift from nutrient mineralization to survival maintenance, leading to functional decline and reduced crop productivity. Pot and field experiments were established with three N application levels (0%, 20%, and 40% reduction) combined with B+M amendments, using conventional fertilization without amendments as the control. By analyzing rice yield formation, soil nutrient content, and bacterial community structure, this study aimed to elucidate the impacts and underlying microbiologically mediated mechanisms of B+M application under N reduction conditions. The findings are expected to provide a theoretical and practical basis for improving paddy soil health and sustaining rice yield under reduced N input, contributing to the sustainable intensification of rice production.

## 2 Materials and methods

### 2.1 Experimental site and materials

This study included both pot and field experiments. The pot experiment was carried out in 2024 at Henan University of Urban Construction, Pingdingshan City, Henan Province, China. The field experiment was conducted in 2025 in Caozhen Town, Pingdingshan City, Henan Province, China, with wheat as the previous crop. The tested soil in field experiment was a silty clay loam paddy soil with a pH of 7.9, containing total N 1.5 g/kg, cation exchange capacity 19.9 cmol/kg, available N 99.5 mg/kg, available phosphorus (P) 21.5 mg/kg, available potassium (K) 75.5 mg/kg, and organic matter 26.2 g/kg. The rice cultivar Nanjing 9108 was used in all trials.

### 2.2 Experimental design

Sowing was conducted on May 25, 2024 and 2025, using tray seedling cultivation with 120 g (dry seeds) per tray. In the pot experiment, each pot was filled with 15 kg of sieved soil, with a pot diameter of 32 cm and height of 26 cm. Transplanting was carried out on June 15, 2024, with 3 hills per pot and 4 seedlings per hill.

Under the condition of combined application of B+M, three treatments were established: conventional N rate N100 (270 kg N/ha), 20% N reduction N80 (216 kg N/ha), and 40% N reduction N60 (162 kg N/ha). A control (CK) with conventional fertilization (270 kg N/ha) but without soil amendments was set up. A zero N control (N0) was also included. The pot experiment was conducted with five treatments (N100, N80, N60, CK, N0), each with three replicates. Each replicate consisted of 20 pots, resulting in a total of 300 pots. In the field experiment, each treatment was replicated three times, resulting in a total of 15 plots. Each plot covered an area of 10 m². To minimize water and nutrient exchange between plots, 40 cm high soil ridges were constructed around each plot and covered with plastic film. The film was buried 30 cm deep into the soil. The biochar (produced by Zhengzhou Haosen Environmental Protection Technology Co., Ltd.) had an organic matter content of 69.7%, an ash content of 9.1%, a moisture content of 5.6%, a total N content of 0.5%, and a total NPK nutrient content of 7.1%. It was produced at a pyrolysis temperature of 400 °C, with a particle size of ≤ 20 mm, and a pH of 6.8. The microbial inoculant was a multi-strain compound agent, including *Azotobacter chroococcum*, *Bacillus subtilis*, and *Bacillus amyloliquefaciens* in powder form, purchased from Beihai Yiqiang Biotechnology Co., Ltd. The application rates were 7.5 t/ha for biochar, and 15 kg/ha, 20 kg/ha, and 20 kg/ha for *Azotobacter chroococcum*, *Bacillus subtilis*, and *Bacillus amyloliquefaciens*, respectively. B+M was applied to the pots and plots one day before rice transplanting.

N fertilizer was applied following a 6:4 split ratio between basal/tillering fertilizer and panicle/grain fertilizer. Specifically, basal and tillering fertilizers were applied in equal amounts during the early stage. Similarly, panicle and grain fertilizers were applied in equal amounts later. Basal fertilizer and tillering fertilizer were applied to the pots one day before transplanting and five days after transplanting, respectively. Panicle fertilizer and grain fertilizer were applied at the 4th leaf age from the top and the 2nd leaf age from the top stages, respectively. The N application rates are detailed in **Table 1**. The application rates of P and K fertilizers were consistent across all treatments. The N: P: K ratio was maintained at 2:1:2 (The ratio is based on pure nutrients (N–P_2_O_5_–K_2_O)). All P and K fertilizers were applied as a basal dose in a single application during pot preparation. Fertilization details for each pot and plot are shown in **Supplementary Tables S1**, **S2**.

**Table 1:** Effects of combined application of biochar and microbial inoculants on rice yield and its components in pot experiment.

Treatment	Panicles number (/pot)	Spikelet panicle^-1^	1000-grain weight (g)	Filled grain (%)	Grain yield (g/pot)
N100	65.3 ± 2.2a	62.6 ± 1.3a	22.3 ± 0.2c	86.0 ± 2.9a	78.3 ± 1.9a
N80	60.8 ± 0.4ab	63.0 ± 4.4a	22.3 ± 0.2c	85.6 ± 1.0a	73.0 ± 5.0ab
N60	55.8 ± 3.4b	64.4 ± 2.8a	22.7 ± 0.2b	82.0 ± 0.2b	66.9 ± 3.8b
CK	63.3 ± 3.5a	58.5 ± 2.6a	23.8 ± 0.1a	79.6 ± 2.2b	70.1 ± 5.4b
η²	0.727	0.444	0.941	0.74	0.533
F	7.1	2.128	42.354	7.599	3.044
df _total_	11	11	11	11	11

Within a column, values followed by the same lowercase letter indicate no significant difference at p< 0.05. N100, N80, and N60 represent nitrogen reduction rates of 0%, 20%, and 40%, respectively, under the combined application of biochar and microbial inoculants. CK represents conventional nitrogen rate with no soil amendments added.

### 2.3 Sampling and measurements

#### 2.3.1 Growth stage recording

The sowing, transplanting, jointing, full heading, and maturity stages were accurately recorded in both pot and field experiment.

#### 2.3.2 Dry matter weight and leaf area index

At the jointing, full heading and maturity stages of both pot and field experiments, three representative hills were selected from each treatment for sampling based on the following criteria: tiller number closest to the treatment mean, plant height within 5% of the mean, and uniform growth stage development. The leaf area per plant was estimated using the length-width coefficient method (leaf area = length × width × 0.75, where 0.75 is the correction coefficient for rice) (Mi et al., 2025). The LAI was then calculated as: LAI = (average leaf area per plant × number of hills per unit area)/ground area. All plant samples were then separated into different organs: leaves, stems (including leaf sheaths), and panicles. These components were placed in an oven at 105°C for 30 minutes for deactivation, then dried at 80°C until a constant weight was achieved to determine the DMW.

#### 2.3.3 Chlorophyll content

At the jointing, full heading, and maturity stages of both pot and field experiments, the topmost fully expanded leaves were collected. Fresh leaf samples (0.1 g) were cut into pieces, placed in a 15 mL centrifuge tube containing 10 mL of 80% (v/v) acetone extraction solution, and kept in the dark for 3 hours. During this period, the tubes were gently shaken for 30 seconds every half hour. Once the leaf pieces became completely white, the absorbance of the extract at 663 nm and 645 nm was measured using a spectrophotometer to calculate the total chlorophyll content. All extraction procedures were carried out under dim light to prevent chlorophyll degradation. Samples were kept at 4 °C throughout the extraction process. Total chlorophyll concentrations were calculated using the following formulas (Qian et al., 2022): Total chlorophyll (mg/L) = 20.21 × A_645_ + 8.02 × A_663_.

#### 2.3.4 Root oxidation activity

At the jointing, full heading, and maturity stages of both pot and field experiment, three representative hills were selected from each treatment. Root samples were processed immediately after collection to maintain enzyme activity. The roots were first rinsed clean with running water. A portion of the fresh roots was then used to determine the α-naphthylamine oxidation activity per unit dry root weight (Zhang et al., 2025). The specific procedure was as follows: 1 g of fresh root sample was weighed and placed in a conical flask containing 50 mL of a 20 mg/L α-naphthylamine solution. The flask was shaken at 25°C for 3 hours. Afterward, 2 mL of the solution was extracted from the flask. Then 1 mL of 1% sulfanilic acid and 1 mL of a 0.1 g/L sodium nitrite solution were added and mixed thoroughly. After color development, the α-naphthylamine content was measured using a spectrophotometer. The oxidation rate was calculated as: Root oxidation activity = [(C_0_ – C_1_) × V]/(FW × t). Where C_0_ and C_1_ are the initial and final α-NA concentrations (μg/mL), V is the solution volume (mL), FW is the fresh root weight (g), and t is the incubation time (h).

#### 2.3.5 Rice yield and its components

At maturity of both pot and field experiments, 10 randomly selected hills from each plot were used for yield component analysis. All panicles from these hills were placed in mesh bags, air dried, and then threshed. The total number of grains from the 10 hills was counted to calculate the number of grains per panicle. Unfilled grains were separated using a water flotation method to determine the filled grain rate. The filled grain rate was calculated as the percentage of filled spikelet to the total number of spikelet: Filled grain rate (%) = (Number of filled spikelet/Total number of spikelet) × 100. The 1000-grain weight was determined by weighing three replicates of 1000 filled grains each (with an error margin not exceeding 0.05 g). The actual grain yield for each pot and plot was determined by harvesting and threshing all plants separately. The harvested grain samples were retained for subsequent rice quality analysis.

#### 2.3.6 Soil chemical properties and microbial diversity

At the maturity stage of both pot and field experiments, destructive sampling was conducted. Rice plants and soil were separated. The soil was passed through a 2 mm sieve and divided into two portions: one portion was immediately stored at -80°C for subsequent analysis of soil microbial diversity; the other portion was air dried naturally for the determination of soil available N, available P, and available K contents. The samples were analyzed following standard soil agrochemical methods: available N content was determined using the alkali hydrolysis diffusion method (Wang et al., 2017); available P content was measured using the sodium bicarbonate extraction method; and available K content was determined using the ammonium acetate extraction method (Li et al., 2019).

Total microbial DNA was extracted from soil samples in the pot experiment using the HiPure Soil DNA Kit (D3142, Guangzhou Meiji Biotechnology Co., Ltd.). The V3-V4 region of the bacterial 16S rRNA gene was amplified using specific primers with barcodes. The primer sequences were: 341F (CCTACGGGNGGCWGCAG) and 806R (GGACTACHVGGG-TATCTAAT). All PCR reactions were carried out in 30 μL reactions with 15μL of Phusion^®^High-Fidelity PCR Master Mix (New England Biolabs); 0.2 μM of forward and reverse primers, and about 10 ng template DNA. Thermal cycling consisted of initial denaturation at 98°C for 1 min, followed by 30 cycles of denaturation at 98°C for 10 s, annealing at 50°C for 30 s, and elongation at 72°C for 60 s. Take an equal volume of 1× loading buffer containing SYBR Green, mix with the PCR products, and perform electrophoresis on a 2% agarose gel for detection. Samples with bright main strip between 400-450bp were chosen for further experiments. The amplified products were quantified using a QuantiFluor™ fluorometer. Purified amplicons were ligated with sequencing adapters to construct sequencing libraries, which were then sequenced on an Illumina PE250 platform. The raw sequencing data have been deposited in the NCBI Sequence Read Archive (SRA) under accession number PRJNA1434724. The raw 16S rRNA gene sequencing reads were demultiplexed, quality-filtered, and processed using the following criteria: (i) Reads were truncated at any site receiving an average quality score< 20 over a 10 bp sliding window, and truncated reads shorter than 50 bp were discarded; reads containing ambiguous characters were also discarded; (ii) Paired-end reads were merged based on their overlapping sequences, with a minimum overlap length of 10 bp; (iii) The maximum mismatch ratio allowed in the overlap region was 0.2, and reads that could not be assembled were discarded; (iv) Samples were distinguished according to their barcodes and primer sequences, with exact barcode matching (0 mismatches) and up to 2 mismatches allowed in primer sequences; sequence direction was adjusted accordingly. In total, 1,135,452 (476,117,286 bp) high quality reads for 16S rRNA genes were obtained. After preprocessing all the reads, the numbers of sequences among different samples ranged from 91643 to 97263 in 16S rRNA genes (**Supplementary Table S3**). The sequencing depth was evaluated by Good’s coverage estimator, which ranged from 0.98 to 0.99 across all samples, indicating that the sequencing depth was sufficient to capture the majority of bacterial diversity (The quality filtering was shown in **Supplementary Figure S1**). The obtained sequencing data were processed using Uparse software (version 9.2.64) to cluster operational taxonomic units (OTUs) at a 97% similarity threshold. Chimeric sequences were identified and removed using the Uchime software. The most abundant sequence within each OTU cluster was selected as the representative sequence. Taxonomic assignment for each OTU was performed using the RDP classifier based on the SILVA reference database (version 132).

### 2.4 Data analysis

Data on rice photosynthetic performance, root activity, aboveground dry matter, and yield components were processed and plotted using Microsoft Excel 2019 and SigmaPlot 11.0 (Systat Software Inc., San Jose, CA, USA). Statistical analyses for these agronomic traits were conducted with SPSS version 27.0 (IBM Corp., Armonk, NY, USA). Data were first tested for normality and homogeneity of variances using the Shapiro–Wilk test and Levene’s test, respectively. One−way analysis of variance (ANOVA) was then applied to evaluate the effects of different treatments on the measured parameters. When ANOVA indicated significant differences (P< 0.05), means were compared using the least significant difference (LSD) multiple comparison test.

Soil microbial community analyses were performed using R software (version 4.5.2, R Foundation for Statistical Computing, Vienna, Austria). Alpha diversity (ACE, Chao1, Shannon, and Simpson) index was calculated for each sample using the vegan package in the R environment (version 4.5.2). Beta diversity was evaluated via principal coordinate analysis (PCoA) based on the Bray–Curtis dissimilarity matrix, using the “ordinate” function in the phyloseq package of R software. A structural equation model (SEM) was carried out using the lavaan package to evaluate causal pathways among variables. And goodness-of-fit indices (χ², df, RMSEA and CFI) were used to evaluate model adequacy. Pearson correlation analysis was performed to explore the relationships between microbial community structure and environmental factors. The functional potential of soil bacterial communities was predicted using PICRUSt2 software in conjunction with the KEGG (Kyoto Encyclopedia of Genes and Genomes) metabolic pathway database. Figures related to microbial data were generated using R 4.5.2 and Microsoft PowerPoint 2019 for final layout adjustments.

Relevant calculation formulas are as follows:

(1) $$\text{Dry matter accumulation }\left(\text{kg}\right):\text{DWA}={\text{W}}_{2}-{\text{W}}_{1}$$

(2) $$\text{Nitrogen partial factor productivity }\left(\text{kg}/\text{kg}\right):\;\text{NPFP}=\frac{{\text{Y}}_{N}}{{\text{N}}_{\text{rate}}}$$

(3) $$\text{Nitrogen agronomic efficiency }\left(\text{kg}/\text{kg}\right):\text{NAE}=\frac{{\text{Y}}_{\text{N}}-{\text{Y}}_{0}}{{\text{N}}_{\text{rate}}}$$

(Y_N_ and Y_0_: Grain yield of different N application rates and 0 N control; W_1_ and W_2_ are dry matter weights measured at the two time points).

## 3 Results

### 3.1 Grain yield

Grain yield varied significantly among treatments (**Tables 1**, **2**). Compared with CK, the N80 and N100 treatments increased grain yield by 4.1% and 11.7%, respectively, whereas the N60 treatment resulted in a 4.6% yield reduction in the pot experiment. Similarly, in the field experiment, the N80 and N100 treatments increased grain yield by 4.9% and 10.4% (p<0.05), respectively, whereas the N60 treatment resulted in a 3.9% yield reduction compared with CK. Yield component analysis revealed that panicle number in both N80 and N100 treatments did not differ significantly from CK in the pot experiment. In the field experiment, however, N80 showed no significant difference from CK, whereas N100 exhibited a significant increase compared to CK. In contrast, the number of spikelet per panicle increased by 7.6% and 7.0% in the pot experiment, and by 1.9% and 3.1% in the field experiment for N80 and N100, respectively. Similarly, the filled grain rate rose by 7.5% (p<0.05) and 8.1% (p<0.05) in pots, and by 2.1% (p<0.05) and 1.5% (p<0.05) in the field. In the N60 treatment, the panicle number decreased by 11.8% (p<0.05) in the pot experiment and 10.3% (p<0.05) in the field experiment. These results indicated that the yield increase under N100 and N80 treatments was driven by different yield components across experiments. In the pot experiment, enhanced spikelet per panicle and filled grain rate contributed to the yield improvement. In the field experiment, the yield increase under N100 resulted from increased panicle number, spikelet per panicle, and filled grain rate, whereas N80 primarily enhanced spikelet per panicle and filled grain rate. In contrast, the low panicle number in the N60 treatment was the main factor leading to its yield decline.

**Table 2:** Effects of combined application of biochar and microbial inoculants on rice yield and its components in field experiment.

Treatment	Panicles number (10^4^/ha)	Spike panicle^-1^	1000-grain weight (g)	Filled grain (%)	Grain yield (t/ha)
N100	370.1 ± 3.7a	106.3 ± 2.8a	26.1 ± 0.4a	96.7 ± 0.5a	9.9 ± 0.3a
N80	348.4 ± 7.9b	107.6 ± 3.8a	26.3 ± 0.1a	96.1 ± 0.1a	9.5 ± 0.5ab
N60	315.4 ± 5.6c	109.3 ± 1.3a	26.3 ± 0.3a	95.3 ± 0.3b	8.6 ± 0.1b
CK	351.5 ± 3.0b	104.3 ± 2.0a	26.5 ± 0.7a	94.7 ± 0.3b	9.1 ± 0.4b
η²	0.953	0.413	0.838	0.878	0.76
F	53.669	1.879	13.798	19.251	8.442
df_total_	11	11	11	11	11

Within a column, values followed by the same lowercase letter indicate no significant difference at p< 0.05. N100, N80, and N60 represent nitrogen reduction rates of 0%, 20%, and 40%, respectively, under the combined application of biochar and microbial inoculants. CK represents conventional nitrogen rate with no soil amendments added.

### 3.2 Dry matter weight

With a reduction in N application rate, the DMW at different growth stages and phases showed a trend of N100>N80>N60 (**Tables 3**, **4**). Compared to CK, the N100 treatment increased DMW by 7.9%, 8.0%, and 11.7% in the pot experiment, and increased by 8.3%, 9.3% and 8.8% in the field experiment at the jointing, full heading, and maturity stages, respectively. The DMA (Equation 1) in the N100 treatment from sowing to jointing, jointing to heading, and heading to maturity increased by 7.9%, 8.1%, and 24.7% in the pot experiment, and increased by 8.3%, 9.7% and 8.1% in the field experiment, respectively, compared to CK. Compared to CK, the DMW in N80 treatment resulted in a 0.1% and 3.8% increase in the pot experiment and 4.2% and 4.5% increase in the field experiment at the full heading and maturity stages, respectively, and enhanced DMA by 1.3% and 16.5% in the pot experiment, and by 6.1% and 5.0% in the field experiment during the jointing to full heading and full heading to maturity phases. In contrast, the N60 treatment decreased DMW by 17.6%, 7.6%, and 7.6% in the pot experiment and decreased by 6.6%, 8.9%, and 6.7% in the field experiment at the jointing, full heading, and maturity stages, respectively, and reduced DMA during the sowing to jointing and full heading to maturity periods by 17.6% and 9.9% in the pot experiment, and by 9.8% and 2.7% in the field experiment, respectively.

**Table 3:** Effects of combined application of biochar and microbial inoculants on dry matter weight in pot experiment.

Treatment	Dry matter weight (g/pot)					
	Jointing stage	Full heading stage	Maturity stage	Seeding to Jointing	Jointing to full heading	Full heading to maturity
N100	56.5 ± 2.1a	127.2 ± 6.0a	168.8 ± 4.1a	56.5 ± 2.1a	70.7 ± 4.9a	41.6 ± 1.8a
N80	51.7 ± 3.6ab	117.9 ± 6.5ab	156.8 ± 5.5ab	51.7 ± 3.6ab	66.2 ± 3.7a	38.9 ± 2.0a
N60	43.1 ± 2.4b	108.7 ± 3.3b	138.8 ± 4.7b	43.1 ± 2.4b	65.6 ± 4.2a	30.1 ± 2.9a
CK	52.4 ± 4.6ab	117.7 ± 8.4ab	151.1 ± 6.7ab	52.4 ± 4.6ab	65.4 ± 5.0a	33.4 ± 1.8a
η²	0.498	0.615	0.489	0.498	0.087	0.115
F	2.646	4.256	2.552	2.646	0.255	0.348
df_total_	11	11	11	11	11	11

Within a column, values followed by the same lowercase letter indicate no significant difference at the 5% probability level according to LSD test. N100, N80, and N60 represent nitrogen reduction rates of 0%, 20%, and 40%, respectively, under the combined application of biochar and microbial inoculants. CK represents conventional nitrogen rate with no soil amendments added.

**Table 4:** Effects of combined application of biochar and microbial inoculants on dry matter weight in field experiment.

Treatment	Dry matter weight (t/ha)					
	Jointing stage	Full heading stage	Maturity stage	Seeding to Jointing	Jointing to full heading	Full heading to maturity
N100	3.9 ± 0.05a	13.0 ± 0.09a	20.0 ± 0.22a	3.9 ± 0.05a	9.2 ± 0.12a	7.0 ± 0.29a
N80	3.5 ± 0.14b	12.4 ± 0.03b	19.2 ± 0.05b	3.5 ± 0.14b	8.9 ± 0.11a	6.8 ± 0.06ab
N60	3.3 ± 0.13c	10.9 ± 0.21d	17.2 ± 0.12d	3.3 ± 0.13c	7.5 ± 0.34c	6.3 ± 0.22b
CK	3.6 ± 0.09b	11.9 ± 0.10c	18.4 ± 0.03c	3.6 ± 0.09b	8.4 ± 0.09b	6.5 ± 0.12b
η²	0.824	0.983	0.99	0.824	0.937	0.752
F	12.444	156.881	259.495	12.444	39.959	8.098
df_total_	11	11	11	11	11	11

Within a column, values followed by the same lowercase letter indicate no significant difference at the 5% probability level according to LSD test. N100, N80, and N60 represent nitrogen reduction rates of 0%, 20%, and 40%, respectively, under the combined application of biochar and microbial inoculants. CK represents conventional nitrogen rate with no soil amendments added.

### 3.3 Leaf and root physiological activity

#### 3.3.1 Leaf photosynthetic capacity

With a reducing N application rate, the LAI under different B+M treatments followed the order N100>N80>N60 (**Table 5**). Compared with CK, the LAI of the N100 treatment increased by 9.2%, 8.6%, and 25.5% in the pot experiment and increased by 23.0%, 12.0%, and 20.2% in the field experiment at the jointing, full heading, and maturity stages, respectively. Corresponding increases for the N80 treatment were 1.3%, 0.7%, and 1.7% in the pot experiment and 5.0%, 3.8% and 7.6% in the field experiment. Conversely, the N60 treatment decreased LAI by 7.7%, 7.1%, and 27.8% in pot experiment, and decreased by 10.0%, 4.4% and 17.7% in the field experiment at these stages.

**Table 5:** Effects of combined application of biochar and microbial inoculants on dry matter accumulation in pot and field experiment.

Treatment		LAI (10^4^ m^2^/ha)		
		Jointing stage	Full heading stage	Maturity stage
Pot experiment	N100	5.9 ± 0.4a	8.0 ± 0.4a	1.8 ± 0.2a
	N80	5.5 ± 0.5a	7.4 ± 0.8ab	1.5 ± 0.1a
	N60	5.0 ± 1.3a	6.8 ± 0.4b	1.0 ± 0.2a
	CK	5.4 ± 0.2a	7.3 ± 0.5ab	1.4 ± 0.1a
	η²	0.223	0.45	0.395
	F	0.764	2.182	1.74
	df_total_	11	11	11
Field experiment	N100	4.1 ± 0.09a	8.2±0.02a	2.9 ± 1.46a
	N80	3.5 ± 0.07b	7.6 ± 0.14b	2.6 ± 1.29b
	N60	3.0 ± 0.10c	7.0 ± 0.14d	2.0 ± 0.11c
	CK	3.3 ± 0.04c	7.3 ± 0.07c	2.4 ± 0.12b
	η²	0.974	0.964	0.986
	F	100.009	71.779	191.266
	df_total_	11	11	11

Within a column, values followed by the same lowercase letter indicate no significant difference at the 5% probability level according to LSD test. N100, N80, and N60 represent nitrogen reduction rates of 0%, 20%, and 40%, respectively, under the combined application of biochar and microbial inoculants. CK represents conventional nitrogen rate with no soil amendments added.

**Figure 1:**
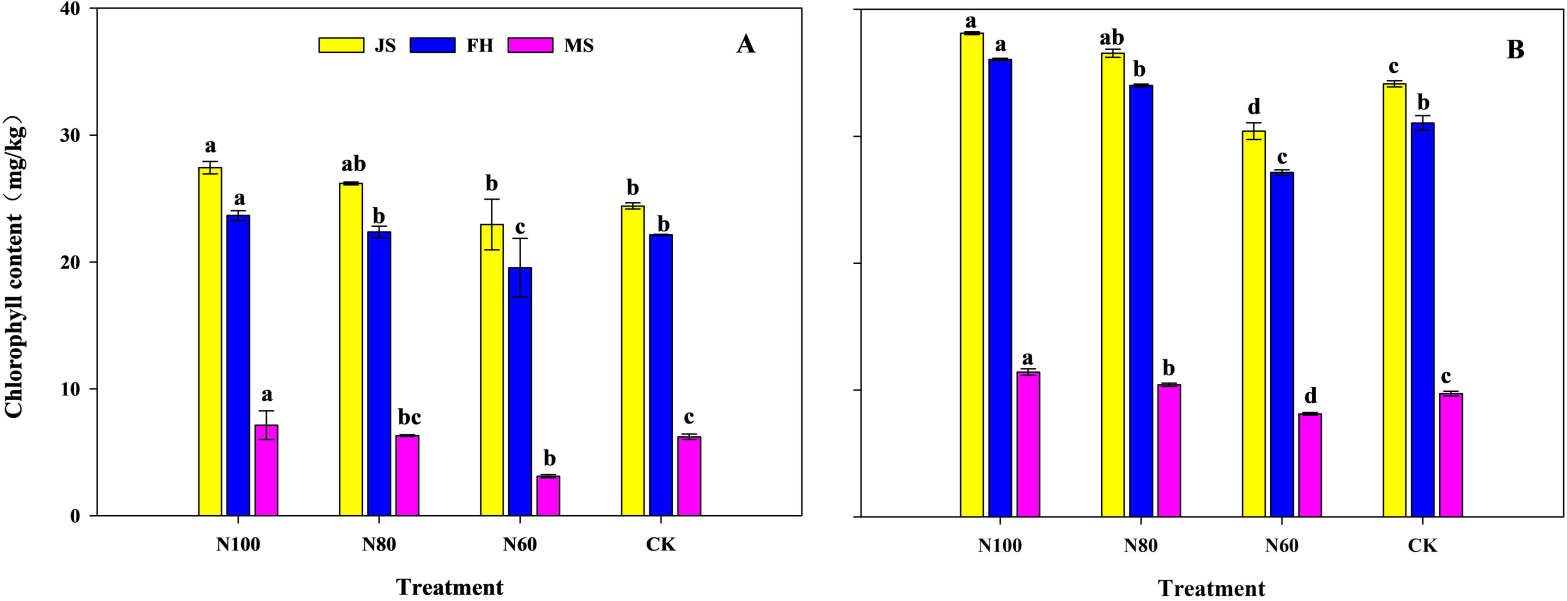
Effects of combined application of biochar and microbial inoculants on chlorophyll content in pot experiment **(A)** and field experiment **(B)**. JS, FH, and MS represent the jointing stage, full heading stage, and maturity stage, respectively. N100, N80, and N60 denote nitrogen reduction rates of 0%, 20%, and 40% under the combined application of biochar and microbial inoculants. CK represents conventional nitrogen rate with no soil amendments added. Within the same growth stage, values followed by the same lowercase letter indicate no significant difference at the 5% probability level according to LSD test.

The chlorophyll content in leaves at different growth stages gradually decreased with a reduction in N application rate under the combined B+M treatments (**Figure 1**). Compared to CK, the N100 treatment increased chlorophyll content by 12.4%, 6.9%, and 14.7% in the pot experiment and increased by 11.7%, 16.1%, and 17.5% in the field experiment at the jointing, full heading, and maturity stages, respectively. Compared to CK, the N80 treatment showed increases of 7.3%, 1.0%, and 1.6% in the pot experiment and increases of 7.0%, 9.6%, and 7.2% in the field experiment at the jointing, full heading, and maturity stages, respectively. In contrast, the N60 treatment decreased chlorophyll content by 6.0%, 11.7%, and 50.0% in the pot experiment and by 10.9%, 12.6%, and 16.5% in the field experiment at the jointing, full heading, and maturity stages.

#### 3.3.2 Root oxidation activity

**Figure 2:**
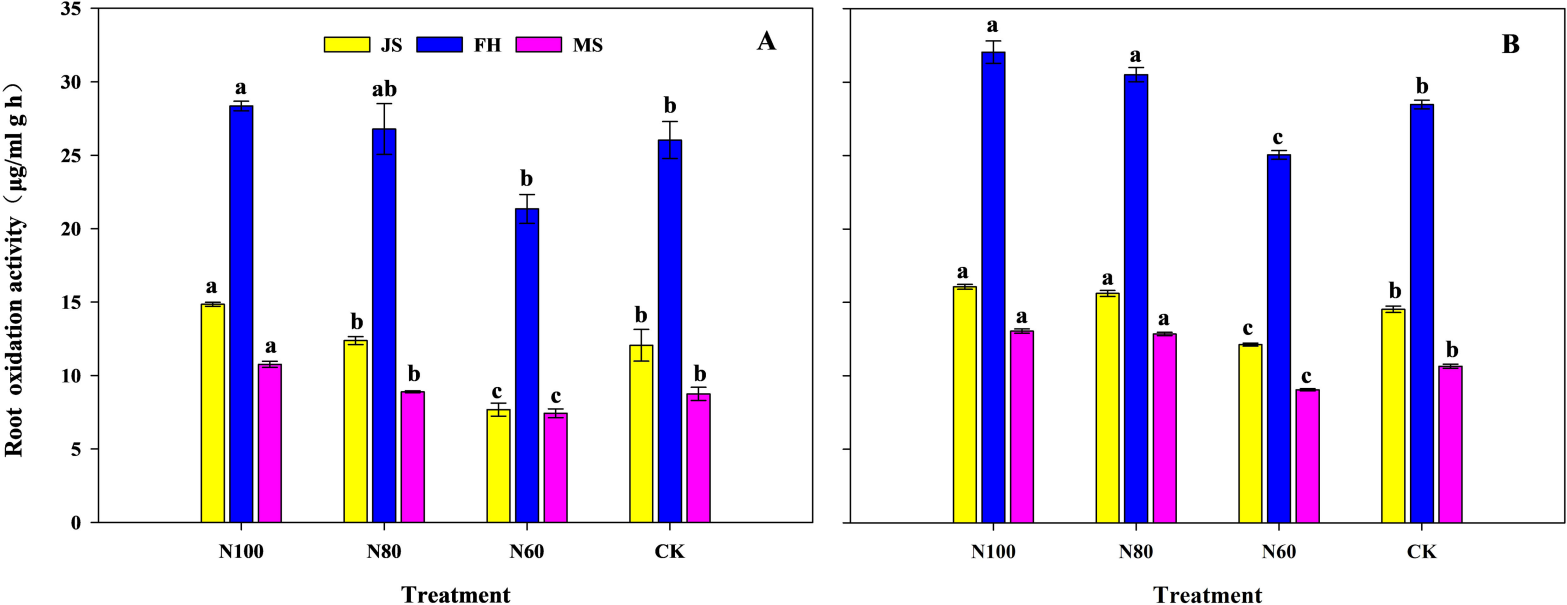
Effects of combined application of biochar and microbial inoculants on root oxidation activity in pot experiment **(A)** and field experiment **(B)**. JS, FH, and MS represent the jointing stage, full heading stage, and maturity stage, respectively. N100, N80, and N60 denote nitrogen reduction rates of 0%, 20%, and 40% under the combined application of biochar and microbial inoculants. CK represents conventional nitrogen rate with no soil amendments added. Within the same growth stage, values followed by the same lowercase letter indicate no significant difference at the 5% probability level according to LSD test.

ROA at different growth stages consistently exhibited the trend of N100>N80>N60 (**Figure 2**). Within the same growth period, ROA in the N100 and N80 treatments was significantly higher than that in CK, while it was lower in the N60 treatment. Specifically, compared to CK, ROA in the N100 treatment increased by 23.1%, 8.9%, and 23.0% in the pot experiment and increased by10.5%, 12.5%, and 22.4% in the field experiment at the jointing, full heading, and maturity stages, respectively. The N80 treatment showed increases of 2.7%, 2.9%, and 1.6% in the pot experiment and increases of 7.5%, 7.2%, and 20.6% in the field experiment at these stages. Conversely, ROA in the N60 treatment decreased by 36.3%, 18.0%, and 15.1% in the pot experiment and by 16.5%, 12.0%, and 15.1% in the field experiment at the jointing, full heading, and maturity stages, respectively.

### 3.4 Nitrogen use efficiency

**Figure 3:**
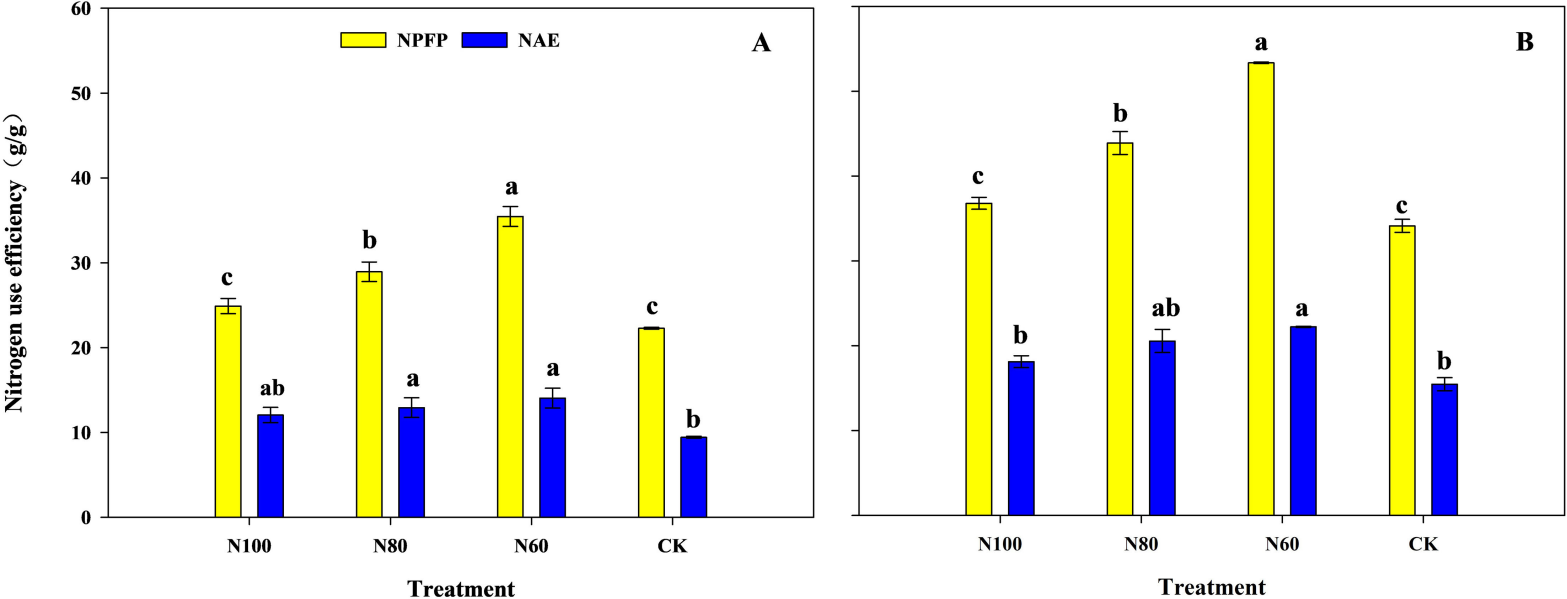
Effects of combined application of biochar and microbial inoculants on nitrogen use efficiency in pot experiment **(A)** and field experiment **(B)**. NPFP and NAE represent the nitrogen partial factor productivity and nitrogen agronomic use efficiency. N100, N80, and N60 denote nitrogen reduction rates of 0%, 20%, and 40% under the combined application of biochar and microbial inoculants. CK represents conventional nitrogen rate with no soil amendments added. Within the same growth stage, values followed by the same lowercase letter indicate no significant difference at the 5% probability level according to LSD test.

With the reduction in N application rate, both the NPFP (Equation 2) and NAE (Equation 3) under the combined B+M treatments gradually increased, and were consistently higher than those in the CK (**Figure 3**). Compared to CK, the N100, N80, and N60 treatments increased NPFP by 11.7%, 30.0% (p<0.05), and 59.3% (p<0.05) in the pot experiment and by 7.8%, 28.6% (p<0.05), and 56.3% (p<0.05) in the field experiment. Compared to CK, the N100, N80, and N60 treatments increased NAE by 27.7%, 37.0% (p<0.05), and 48.9% (p<0.05) in the pot experiment and by 17.2%, 33.0%, and 43.8% (p<0.05) in the field experiment, respectively.

### 3.5 Soil nutrient content

**Figure 4:**
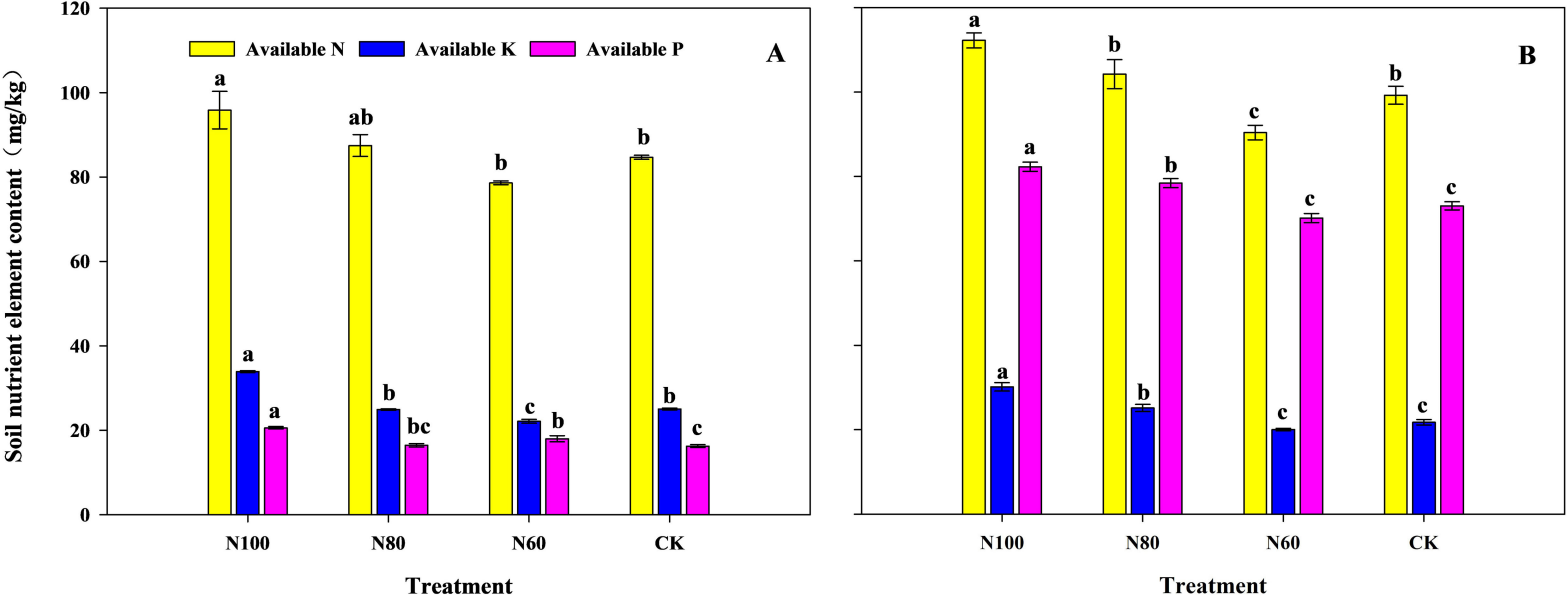
Effects of combined application of biochar and microbial inoculants on the soil nutrient content in pot experiment **(A)** and field experiment **(B)**. N100, N80, and N60 denote nitrogen reduction rates of 0%, 20%, and 40% under the combined application of biochar and microbial inoculants. CK represents conventional nitrogen rate with no soil amendments added. Within the same growth stage, values followed by the same lowercase letter indicate no significant difference at the 5% probability level according to LSD test.

The contents of available N, available P, and available K in the soil generally decreased with a reduction in N application rate, except for the available P in the N60 treatment of the pot experiment (**Figure 4**). The soil available N, P and K contents in the N100 and N80 treatments were higher than those in the CK (except for the K content of N80 in the pot experiment), whereas the available N, P and K contents in the N60 treatment were lower than those in the CK (except for the P content of N60 in the pot experiment). Notably, the differences between the CK and the N100 treatment reached significant levels for all measured nutrients. Compared to the CK, the N80 treatment increased available N and P by 3.3% and 1.4% in the pot experiment and increased available N, K and P by 5.0% (p<0.05), 15.6% (p<0.05) and 7.4% (p<0.05) in the field experiment, respectively. In contrast, the N60 treatment decreased the contents of N and K by 7.1% and 11.6% (p<0.05) in the pot experiment and decreased N, K and P contents by 8.9% (p<0.05), 8.0% and 4.0% in the field experiment, respectively.

### 3.6 Soil bacterial community diversity and structure

#### 3.6.1 Soil bacterial community diversity

**Figure 5:**
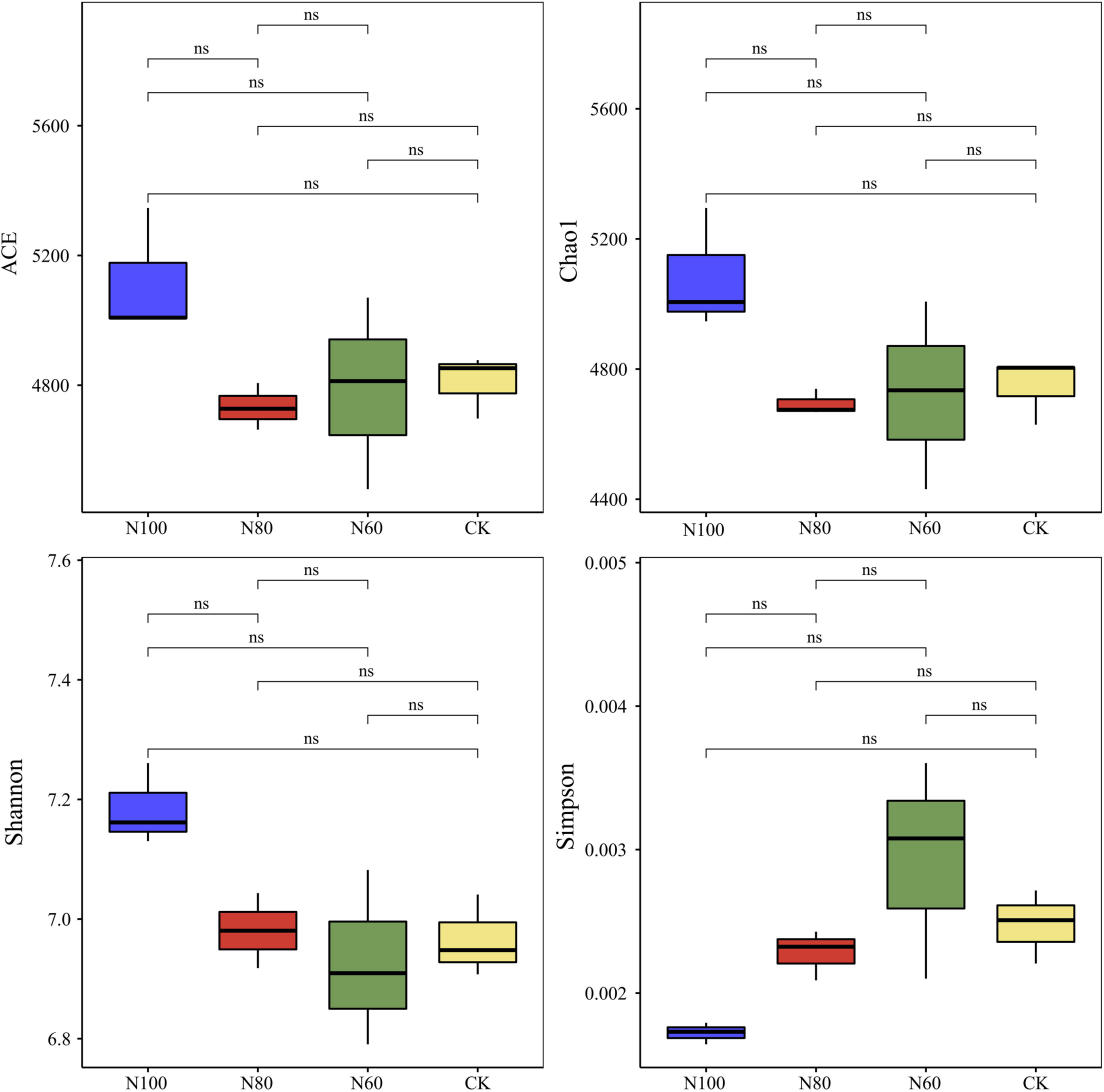
Effects of combined application of biochar and microbial inoculants on alpha diversity of soil bacterial community. N100, N80, and N60 denote nitrogen reduction rates of 0%, 20%, and 40% under the combined application of biochar and microbial inoculants. CK represents conventional nitrogen rate with no soil amendments added. ns (not significant) indicates no significant difference among treatments at the 5% probability level according to LSD test.

In the treatments with combined B+M, with the reduction in N application rate, the ACE and Chao1 indices initially decreased and then increased, while the Shannon index gradually decreased, and the Simpson index progressively increased (**Figure 5**). This pattern suggests a reduction in soil bacterial biodiversity following N reduction, although the differences did not reach statistical significance. Compared to CK, the N100 treatment significantly enhanced soil bacterial diversity, increasing the ACE, Chao1, and Shannon indices by 6.5%, 7.1%, and 3.1%, respectively, while decreasing the Simpson index by 31.1%. The ACE, Chao1, and Simpson indices for the N80 treatment were lower than those for CK, but its Shannon index was higher. For the N60 treatment, the ACE, Chao1, and Shannon indices were all lower than those in CK, while its Simpson index was higher. These results indicated that, compared to conventional fertilization, the combined application of B+M tended to increase soil bacterial diversity under N100 and N80 fertilization conditions, but led to a decline in soil bacterial abundance under the N60 condition.

Principal Coordinate Analysis revealed that PC1 and PC2 accounted for 27.9% and 23.25% of the total variation among different treatments, respectively (**Supplementary Figure S2**). The soil bacterial community structures of the different treatments showed clear separation. The N100 sample points were distinctly distant from those of other treatments, indicating a significant difference in its soil bacterial community composition. Both N80 and N60 were clearly separated from CK, demonstrating that the addition of biochar combined with microbial inoculants significantly altered the soil bacterial community structure under varying N rates (**Supplementary Figure S2**).

#### 3.6.2 Soil bacterial community structure

**Figure 6:**
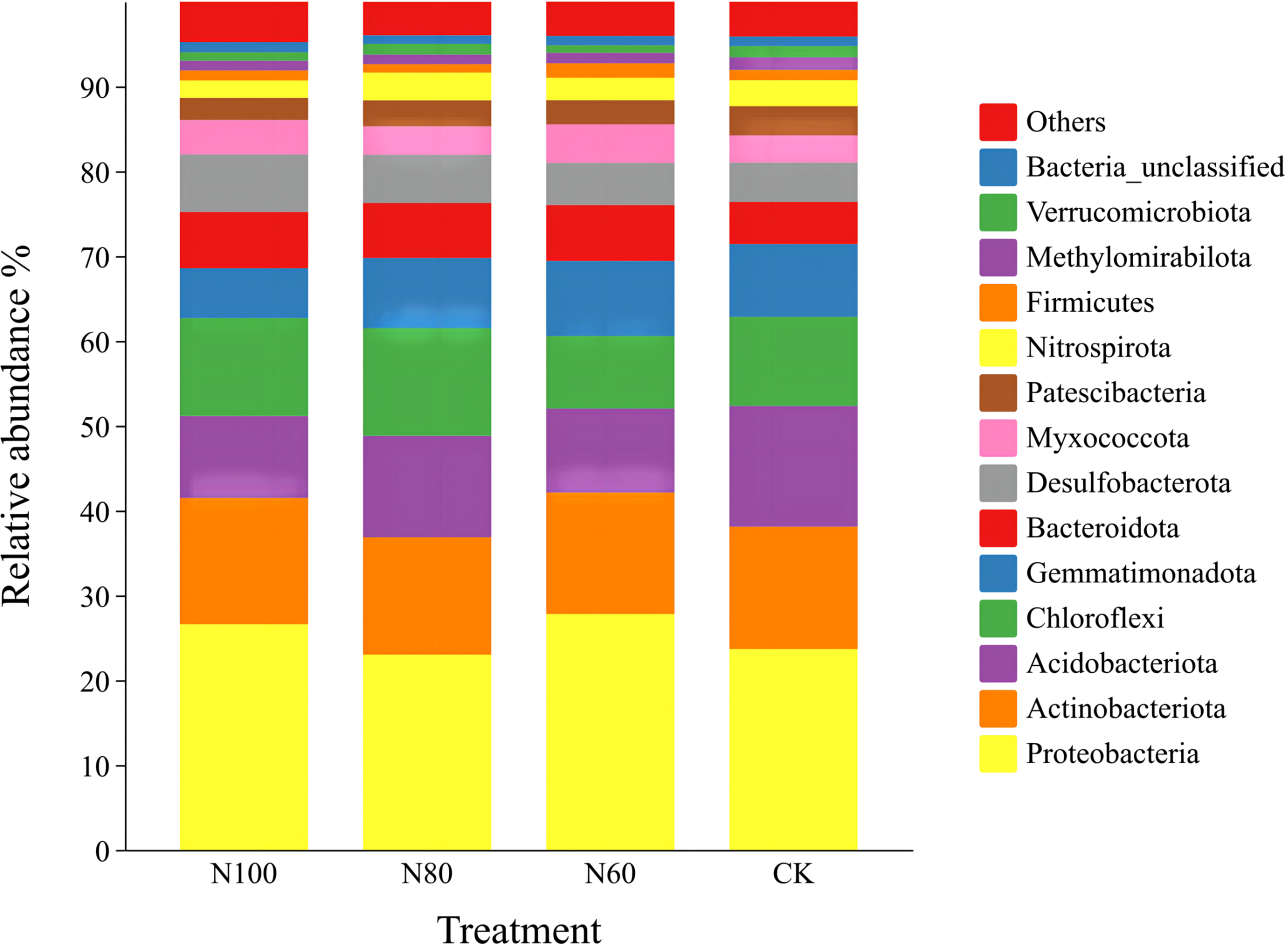
Effects of combined application of biochar and microbial inoculants on phylum-level bacterial community diversity in soil. N100, N80, and N60 denote nitrogen reduction rates of 0%, 20%, and 40% under the combined application of biochar and microbial inoculants. CK represents conventional nitrogen rate with no soil amendments added.

Proteobacteria, Actinobacteria, Acidobacteria, Chloroflexi, Gemmatimonadetes, Bacteroidota, and Desulfobacterota were the dominant phyla across all treatments, collectively accounting for 81.0%-82.0% of the bacterial communities (**Figure 6**). In the B+M treatments, the relative abundances of Actinobacteria, Bacteroidota, and Desulfobacterota decreased to varying degrees with a reduction in N application rate. Specifically, the N100 treatment showed abundances of Actinobacteria, Bacteroidota, and Desulfobacterota that were 7.7%, 2.3%, and 19.0% (p<0.05) higher than those in N80, and 4.1%, 0.5%, and 36.7% (p<0.05) higher than those in N60, respectively. Conversely, the abundances of Acidobacteria and Gemmatimonadetes increased to different extents. The abundances of Acidobacteria and Gemmatimonadetes in the N100 treatment were 19.4% and 28.8% lower than those in N80, and 2.6% and 33.5% (p<0.05) lower than those in N60, respectively. Compared to CK, the N100 treatment increased the relative abundances of Proteobacteria, Actinobacteria, Chloroflexi, Bacteroidota, and Desulfobacterota by 12.3%, 3.3%, 9.8%, 33.3% (p<0.05), and 46.2% (p<0.05), respectively, but decreased the abundances of Acidobacteria and Gemmatimonadetes by 32.1% and 31.3%. The N80 treatment resulted in increased abundances of Chloroflexi, Bacteroidota, and Desulfobacterota, while the abundances of other phyla decreased compared to CK. In the N60 treatment, the abundances of Actinobacteria, Acidobacteria, and Chloroflexi decreased by 0.8%, 30.4% (p<0.05) and 18.6%, whereas the abundances of the remaining bacterial phyla increased.

**Figure 7:**
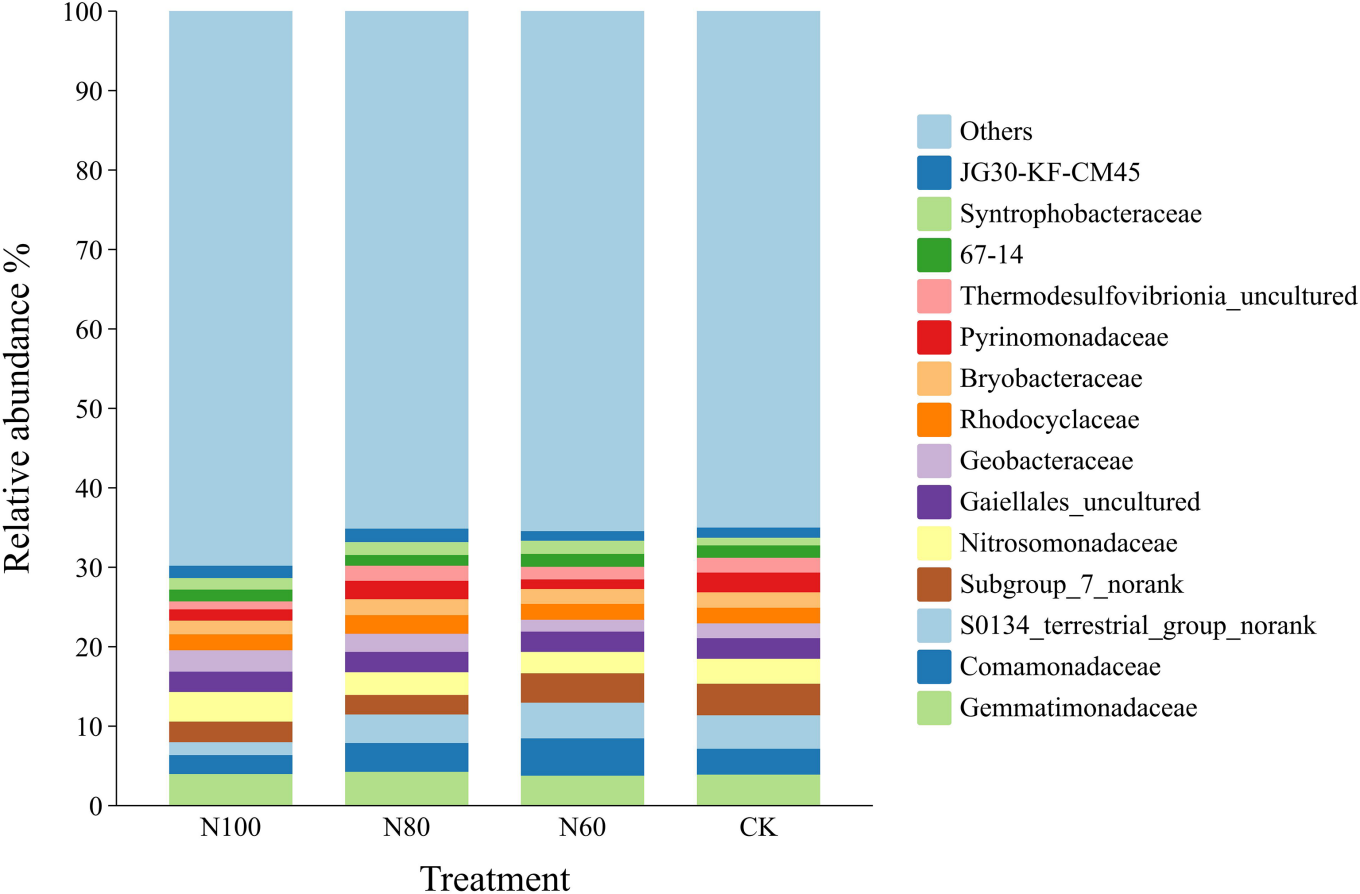
Effects of combined application of biochar and microbial inoculants on family-level bacterial community diversity in soil. N100, N80, and N60 denote nitrogen reduction rates of 0%, 20%, and 40% under the combined application of biochar and microbial inoculants. CK represents conventional nitrogen rate with no soil amendments added.

Gemmatimonadaceae, Comamonadaceae, S0134_terrestrial_group_norank, Subgroup_7_norank, Nitrosomonadaceae, Gaiellales_uncultured, and Geobacteraceae were the dominant families across all treatments (**Figure 7**). In the combined B+M treatments, the abundances of Comamonadaceae and S0134_terrestrial_group_norank gradually increased with a reduction in N application rate, while the abundances of Nitrosomonadaceae and Geobacteraceae gradually decreased. Specifically, in the N60 treatment, the abundances of Comamonadaceae and S0134_terrestrial_group_norank were 95.0% and 180.6% higher than those in the N100 treatment, respectively, whereas the abundances of Nitrosomonadaceae and Geobacteraceae were 27.7% and 43.7% lower than those in N100. Compared to CK, the N100 treatment increased the abundances of Gemmatimonadaceae, Nitrosomonadaceae, and Geobacteraceae by 1.5%, 18.8%, and 42.1%, respectively, while the abundances of other bacterial families decreased. The N80 treatment resulted in increased abundances of Gemmatimonadaceae, Comamonadaceae, and Geobacteraceae by 8.4%, 12.3%, and 20.0%, respectively, with decreases observed in the remaining families. In the N60 treatment, only the abundances of Comamonadaceae and S0134_terrestrial_group_norank increased, while the abundances of all other families decreased.

### 3.7 Correlation analysis between soil bacterial community structure and soil physicochemical properties

**Figure 8:**
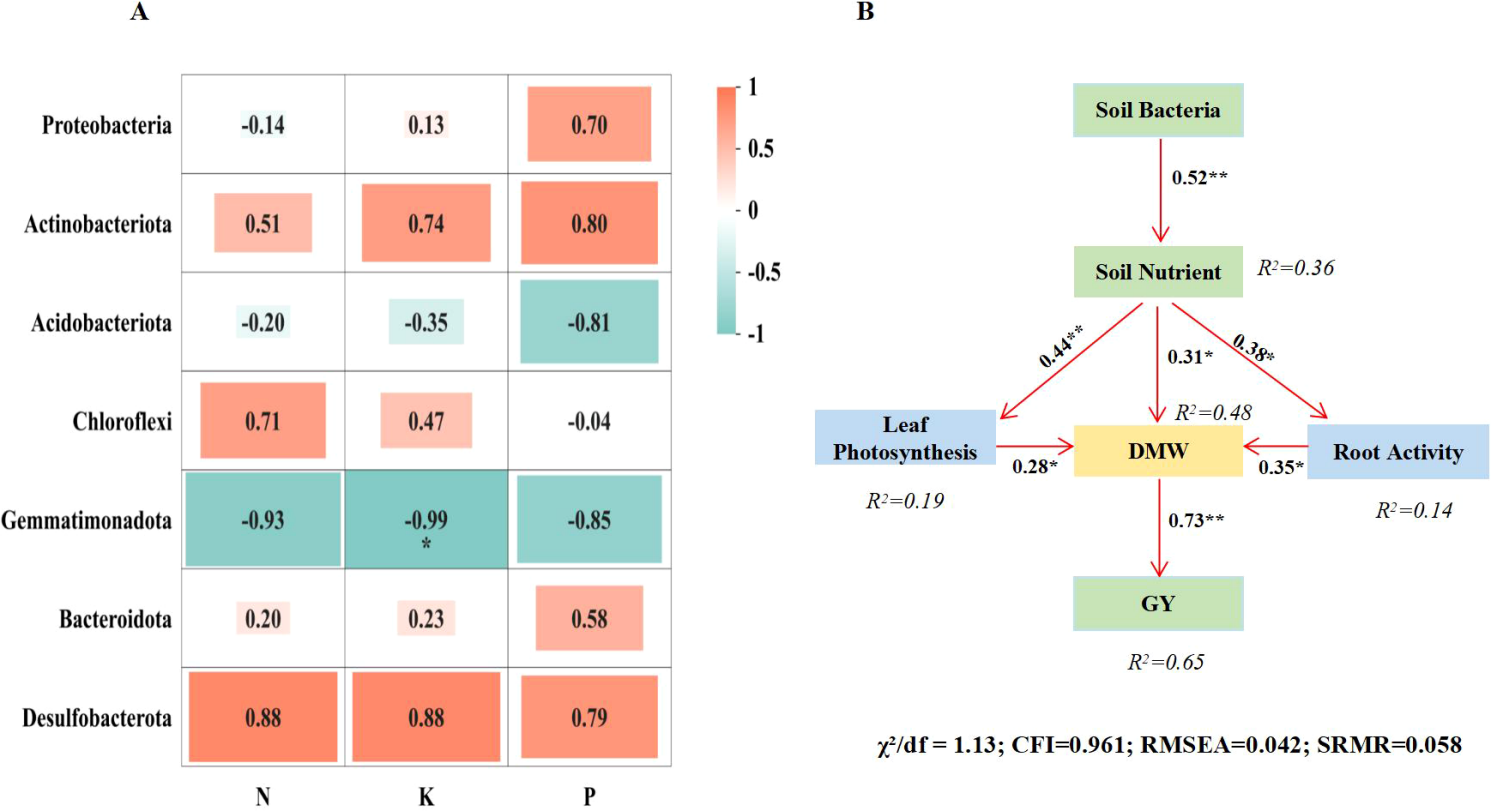
Correlation analysis between soil nutrients and the abundance of dominant bacterial phyla **(A)** and Structural equation modeling (SEM) illustrating the effects of soil properties on rice yield **(B)**. * and ** indicate significance levels at P<0.05 and P<0.01, respectively; red connections represent positive relationships; values on the connections denote standardized path coefficients.

The phyla Desulfobacterota, Bacteroidota, and Actinobacteria were positively correlated with the contents of soil available N, P and K (**Figure 8A**). The phylum Chloroflexi showed positive correlations with soil available P and K, while Proteobacteria was positively correlated with soil available P. A SEM was constructed to test the hypothesized pathways linking soil bacterial diversity to rice yield via soil nutrients and plant physiological traits (**Figure 8B**). Soil bacterial community had a significant positive effect on soil available nutrients (β = 0.52, P = 0.003). Soil nutrients, in turn, positively influenced leaf photosynthesis (β = 0.44, P = 0.008), root activity (β = 0.38, P = 0.012), and directly increased dry matter accumulation (β = 0.31, P = 0.023). Both photosynthesis (β = 0.28, P = 0.041) and root activity (β = 0.35, P = 0.018) further contributed to dry matter accumulation, which ultimately drove grain yield (β = 0.73, P< 0.001). The model explained 65% of the variance in grain yield, confirming that the positive effect of bacterial community on yield is primarily mediated through enhanced nutrient availability and improved plant physiological performance. These findings indicate that the combined application of B+M promotes rice growth and increases yield by improving the soil bacterial community structure and thereby enhancing soil quality.

### 3.8 KEGG functional prediction

Functional prediction analysis revealed that the combined application of B+M significantly influenced soil bacterial community functions related to Metabolism, Cellular Processes, Genetic Information Processing, and Environmental Information Processing (**Table 6**). With the reducing N application rate, most functional categories showed a gradual decreasing trend, except for metabolism of other amino acids, biodegradation and metabolism of xenobiotics, cell communication, cell growth and death, cell motility, and signal transduction. Significant differences were observed between the N100 treatment and the N80/N60 treatments in functions such as energy metabolism, nucleotide metabolism, metabolism of cofactors and vitamins, replication and repair of genetic information, folding, sorting, and degradation of genetic information, and membrane transport. Compared with N100, these indicators in N60 decreased by 5.1%, 4.4%, 4.5%, 4.0%, 4.3% and 1.6%, respectively.

**Table 6:** Effects of biochar application combined with microbial inoculants on the function of soil bacterial community.

Function level 1	Function level 2	CK	N100	N80	N60	η²	F	df_total_
Metabolism	Amino Acid Metabolism	8416451 ± 273088ab	8582144 ± 226103a	8229237 ± 162865ab	8095449 ± 127422b	0.548	3.234	11
	Biosynthesis of Other Secondary Metabolites	835316 ± 11350a	842858 ± 13425a	816972 ± 26384a	779820 ± 19642b	0.719	6.828	11
	Carbohydrate Metabolism	8059130 ± 250560a	8234829 ± 170657a	7889556 ± 180462ab	7669524 ± 158925b	0.636	4.666	11
	Energy Metabolism	4608770 ± 103226ab	4725075 ± 48233a	4507956 ± 107169b	4374701 ± 58133b	0.782	9.557	11
	Enzyme Families	1558526 ± 23330a	1575175 ± 26636a	1524134 ± 39000ab	1486787 ± 16204b	0.695	6.064	11
	Glycan Biosynthesis and Metabolism	1605884 ± 7568a	1580573 ± 32867a	1557061 ± 47038a	1492907 ± 11468b	0.752	8.083	11
	Nucleotide Metabolism	2457356 ± 4993ab	2497723 ± 45535a	2404427 ± 59224b	2348673 ± 26436b	0.682	5.726	11
	Lipid Metabolism	3082550 ± 109918a	3114946 ± 85138a	3008382 ± 60043a	2976348 ± 55829a	0.416	1.897	11
	Metabolism of Cofactors and Vitamins	3306040 ± 75094ab	3378388 ± 51298a	3231689 ± 75036b	3157800 ± 40009b	0.724	6.991	11
	Metabolism of Other Amino Acids	1466126 ± 65353a	1501591 ± 48170a	1428912 ± 26134a	1434984 ± 22140a	0.392	1.722	11
	Metabolism of Terpenoids and Polyketides	1769206 ± 71544a	1810270 ± 49881a	1728196 ± 32431a	1716511 ± 35663a	0.452	2.196	11
	Xenobiotics Biodegradation and Metabolism	2850982 ± 190414a	2933712 ± 118399a	2772168 ± 67328a	2814221 ± 47507a	0.271	0.991	11
Cellular Processes	Cell Communication	308 ± 28b	466 ± 75a	259 ± 11b	414 ± 29a	0.846	14.7	11
	Cell Growth and Death	431276 ± 16303ab	446354 ± 11106a	419805 ± 5219b	421652 ± 3163b	0.609	4.151	11
	Cell Motility	2641873 ± 103054a	2648529 ± 96199a	2542065 ± 14099a	2554456 ± 61010a	0.374	1.596	11
	Transport and Catabolism	259893 ± 12369a	263252 ± 7239a	251106 ± 6579a	250662 ± 5941a	0.388	1.69	11
Genetic Information	Transcription	1850191 ± 44476a	1857950 ± 41273a	1800592 ± 45446ab	1755504 ± 29021b	0.608	4.144	11
	Translation	3375367 ± 54554a	3425231 ± 62848a	3315286 ± 83972ab	3229147 ± 26049b	0.686	5.829	11
	Replication and Repair	5320572 ± 109991ab	5419145 ± 111185a	5211122 ± 120714b	5105593 ± 49354b	0.666	5.321	11
	Folding, Sorting and Degradation	1785976 ± 33359ab	1818991 ± 27538a	1741259 ± 42671b	1709107 ± 16482b	0.727	7.102	11
Environmental Information Processing	Membrane Transport	8757854 ± 382971ab	9116723 ± 203171a	8659452 ± 103959b	8613738 ± 129704b	0.52	2.892	11
	Signal Transduction	1740892 ± 62299a	1759304 ± 42012a	1685979 ± 22853a	1693803 ± 21553a	0.464	2.307	11
	Signaling Molecules and Interaction	145561 ± 1404a	146714 ± 4247a	143227 ± 4820a	135499 ± 1574b	0.716	6.71	11

Within a column, values followed by the same lowercase letter indicate no significant difference at the 5% probability level according to LSD test. N100, N80, and N60 represent nitrogen reduction rates of 0%, 20%, and 40%, respectively, under the combined application of biochar and microbial inoculants. CK represents conventional nitrogen rate with no soil amendments added.

For metabolism-related functions, compared to CK, the N100 resulted in higher abundances for most KEGG pathways (with the exception of glycan biosynthesis and metabolism), although these increases did not reach statistical significance. Specifically, N100 increased carbohydrate metabolism, energy metabolism, metabolism of cofactors and vitamins, metabolism of other amino acids, metabolism of terpenoids and polyketides, and xenobiotics biodegradation and metabolism by 2.18%, 2.52%, 2.19%, 2.42%, 2.32% and 2.90%, respectively. In contrast, the N60 exhibited significant reduction in biosynthesis of other secondary metabolites (−6.6%, p<0.05), carbohydrate metabolism (−4.8%, p<0.05), enzyme families (−4.6%, p<0.05), and glycan biosynthesis and metabolism (−7.0%, p<0.05) compared to CK. The N80 treatment showed intermediate values, with no significant differences from CK in most metabolic pathways, suggesting functional compensation at 20% N reduction. Genetic information processing followed a similar pattern. Compared to CK, N100 slightly enhanced transcription (+0.4%), translation (+1.5%), replication and repair (+1.9%), and folding, sorting and degradation (+1.9%), though none of these increases were statistically significant. However, N60 significantly reduced transcription (−5.1%, p<0.05) and translation (−4.3%, p<0.05) relative to CK. Again, N80 exhibited intermediate values with no significant differences from CK. For environmental information processing, compared to CK, N100 increased membrane transport, signal transduction, and signaling molecules and interaction by 4.1%, 1.1% and 0.8%, respectively, but none of these increases were statistically significant. Notably, N60 significantly reduced signaling molecules and interaction by 6.9% compared to CK. The N80 treatment showed intermediate values, with no significant differences from CK. Cellular processes exhibited the most striking treatment effect in the cell communication pathway. Compared to CK, N100 significantly increased cell communication by 51.3% (p<0.05) and N60 also showed a significant increase of 34.4%. The N80 treatment did not differ significantly from CK in this pathway.

## 4 Discussion

### 4.1 Effects of combined application of biochar and microbial inoculants on soil bacterial community structure and function

The sole or combined application of biochar and microbial inoculants had a significant impact on the composition of soil bacterial communities. Tang et al. (2021) reported that biochar amendment enhanced microbial diversity and richness in paddy soils, with Acidobacteria, Actinobacteria, Nitrospirae, and Patescibacteria identified as the dominant phyla under biochar treatment. Similarly, Xiao et al. (2022) found that microbial inoculations markedly altered bacterial community structure, with a more pronounced effect on rare bacterial taxa than on abundant ones in paddy soil. Hu and Si (2024) concluded that compared to the sole application of microbial fertilizer, the combined application with biochar significantly increased rice soil bacterial and actinobacterial biomass by 35.9-61.6% and 25.4-39.9%, respectively. The results of our pot experiments indicate that the addition of B+M to rice soil enhanced bacterial community diversity in the N100 treatment compared to CK, with the ACE, Chao1, and Shannon indices increasing by 6.5%, 7.1%, and 3.1%, respectively (**Figure 5**). The abundances of Proteobacteria, Actinobacteria, Chloroflexi, and Bacteroidota were markedly increased in the N100 and N80 soils (**Figure 6**). Proteobacteria and Actinobacteria are dominant phyla in paddy soil, involved in N fixation, disease suppression, and improving soil physicochemical properties (Chaudhry et al., 2012; Sun et al., 2016). Chloroflexi contributes to biogeochemical cycles of C, N, and S (Xian et al., 2020), while Bacteroidota efficiently decomposes labile carbon (Brinkmann et al., 2022). Changes in bacterial abundance subsequently affect nutrient content in paddy soil (Song et al., 2021). The correlation analysis in this study also showed positive correlations between the abundance of key bacterial phyla and the content of soil available N, P, and K in pot experiments (**Figure 8**). This study further elucidates the systemic functional enhancements corresponding to the aforementioned shifts in community structure. Under the N100 treatment, the gene expression potential of the soil microbial community in core functional modules such as metabolism, genetic information processing, environmental information processing, and cellular processes were significantly strengthened (**Table 6**). This functional optimization directly translated into a significant increase in the content of available soil N, P, and K, providing sustained and balanced nutrient supply for rice growth (**Figure 8**).

The synergistic effects observed in this study can be attributed to multiple functional mechanisms of biochar that enhanced microbial inoculant efficacy. First, the highly porous structure of biochar provided protected microhabitats for inoculated microorganisms, facilitating their colonization and persistence against competition from native soil microbiota (Li et al., 2023b; Wu et al., 2025). This physical protection is particularly important in the initial establishment phase following application, explaining the enhanced bacterial diversity in N100 and N80 treatments (**Figure 5**). Second, biochar’s high cation exchange capacity and surface functional groups enhanced nutrient retention, reducing N losses through leaching and volatilization (Gorovtsov et al., 2020). This nutrient retention mechanism likely contributed to the maintained soil available N levels under 20% N reduction (**Figure 4**), effectively buffering against the reduced N input. Third, the labile carbon fractions in biochar served as an energy source for microorganisms, stimulating microbial activity and priming the decomposition of native soil organic matter (Sun et al., 2022). This ‘priming effect’ may have contributed to the enhanced nutrient mineralization observed in N100 and N80 treatments, as indicated by the upregulation of carbohydrate metabolism and amino acid metabolism pathways (**Table 6**).

### 4.2 Effects of combined application of biochar and microbial inoculants on soil nutrient content

High quality soil is fundamental for achieving high crop yields. Numerous studies have shown that B+M could effectively improve soil quality and increase soil nutrient content, with their combined application often yielding superior results compared to their sole application. In rice paddy systems specifically, Hu and Si (2024) reported that the combined application of microbial fertilizer and biochar significantly increased soil organic matter, available N, available P, and available K compared to the sole application of microbial inoculants. Wu et al. (2023) found that, compared to fertilizer-only treatment, the application of B+M increased the total organic carbon content of paddy soil by 10.0% and enhanced soil cation content, but had no effect on soil pH. Our study reached similar conclusions. Compared to CK, the N100 treatment increased the contents of available N, P, and K by 13.2%, 35.5%, and 26.8% in the pot experiment and by 13.1%, 12.7%, and 38.6% in the field experiment, respectively (**Figure 4**).

The observed soil quality under N100 and N80 treatments was closely associated with shifts in soil bacterial community structure and function. Specifically, the increased abundance of Proteobacteria and Actinobacteria (**Figure 6**)-phyla widely recognized for their roles in N cycling-suggests enhanced nitrification and N fixation potential (Ni et al., 2022). Within Proteobacteria, the family Nitrosomonadaceae, which contains ammonia-oxidizing bacteria (Lourenço et al., 2022), showed 18.8% higher abundance in N100 compared to CK (**Figure 7**). This aligns with the increased soil available N content (**Figure 4**) and suggests that B+M application may stimulate ammonia oxidation, the rate limiting step of nitrification (Li et al., 2023a). The enrichment of Bacteroidota (33.3% increase in N100) is particularly significant for N cycling in paddy soils. Bacteroidota are known for their capacity to decompose complex organic polymers, including proteins and chitin, thereby facilitating N mineralization (Brinkmann et al., 2022). This functional trait likely contributed to the increased soil available N observed in N100 and N80 treatments, as organic N mineralization supplemented the inorganic N pool. Functional prediction analysis provided further evidence for enhanced N cycling potential. The significant upregulation of genes associated with amino acid metabolism and energy metabolism in N100 (**Table 6**) suggests increased activity of enzymes involved in N assimilation and transformation. These findings are consistent with previous studies demonstrating that biochar-microbe interactions can enhance N cycling gene abundance (Shanmugam et al., 2021).

### 4.3 Effects of combined application of biochar and microbial inoculants on grain yield

Crop yield is the ultimate manifestation of interactions within the soil-plant system. Previous studies have shown that biochar improves soil physicochemical properties, while the introduction of functional microbial inoculants enhances soil nutrient cycling, providing ample nutrients for crop root systems (Xiang et al., 2023; Chi et al., 2024). Their combined application exhibits a strong yield enhancing effect in rice production (Win et al., 2019; Liu et al., 2017). The results of this study also demonstrate that compared to CK, the N100 treatment significantly increased rice yield by 11.7% in the pot experiment and by 10.4% in the field experiment (**Tables 1** and **2**). This yield increase can be attributed to enhanced ROA (**Figure 2**) and leaf photosynthetic capacity (**Table 5** and **Figure 1**), which led to greater DMW at different growth stages, ultimately resulting in significant yield improvement (**Tables 1** and **2**). These findings are largely consistent with the results reported by Ma et al. (2022) and Win et al. (2019). The enhanced ROA (**Figure 2**) is likely attributable to increased soil available N, which stimulates root growth and metabolic activity (Turino Mattos et al., 2023). The positive correlation between soil available N and ROA (**Figure 8**) supports this interpretation. Enhanced root activity facilitated greater N uptake, as reflected in the substantial improvements in NPFP and NAE. Leaf photosynthetic capacity responses further contributed to yield maintenance. The increased Chlorophyll content (6.9-17.5% in N100 and N80) (**Figure 1**) reflects improved N allocation to photosynthetic machinery, as leaf N content is strongly correlated with chlorophyll concentration (Dusenge et al., 2020). Furthermore, the pot experiment showed that the increased soil available N under the N100 was attributed to the enhanced abundance of N metabolism-related bacteria (Proteobacteria and Actinobacteria) in the soil (Lourenço et al., 2022). KEGG functional prediction also indicated that the expression levels of genes related to amino acid and energy metabolism were significantly higher in the N100 treatment than in CK. SEM analysis also indicates that the fundamental factor behind the yield increase from the combined application of B+M originates from the soil microorganisms (**Figure 8**).

A key finding of this study is the identification of an N-dependent threshold-like pattern governing the synergistic effects of B+M application. While 20% N reduction enhanced yield, 40% reduction led to significant yield losses. For instance, Wei et al. (2021) reported that biochar combined with 25% N reduction maintained maize yield, but 50% reduction caused significant yield losses. This threshold effect can be explained by three interconnected mechanisms operating at the microbial, plant, and ecosystem levels. Soil microbial communities possess functional redundancy-the capacity of multiple taxa to perform similar functions (Louca et al., 2018). However, this redundancy is not unlimited. Under 40% N reduction, the abundance of key functional phyla Actinobacteria, Acidobacteria, and Chloroflexi decreased by 0.8%, 30.4% and 18.6% compared to N100 (**Figure 6**). These phyla are critical for organic matter decomposition, N cycling, and stress tolerance (Wang et al., 2024). Their decline likely reduced the functional capacity of the microbial community, leading to nutrient mineralization could no longer compensate for reduced N input (Wang et al., 2023b). Functional prediction analysis supports this interpretation, showing significant downregulation of multiple metabolic pathways in N60 (**Table 6**). Additionally, ROA declined by 12.0-36.3% across growth stages (**Figure 2**), indicating impaired root metabolic activity and nutrient uptake capacity. Chlorophyll content decreased by 6.0-50.0% (**Figure 1**), reflecting N deficiency-induced chlorosis and reduced photosynthetic capacity. These physiological impairments collectively reduced DMW (-6.6 to -17.6%) and yield (-4.6%) (**Tables 1**, **2**). Furthermore, the 40% N reduction likely exceeded the buffering capacity of the B+M amended soil system. While biochar enhanced nutrient retention and microbial activity stimulated mineralization, these mechanisms could only compensate for a limited reduction in external N input. When the external N supply was reduced excessively, the combined effects of retained soil N, mineralized organic N, and enhanced uptake efficiency were insufficient to meet crop demand (Zhang et al., 2024; Han et al., 2021). Overall, below a threshold, the system shifted from N sufficiency to deficiency, with cascading effects on microbial communities, plant physiology, and yield (Xia et al., 2020). This threshold (216 kg N/ha) can be described as an observed threshold-like pattern rather than a proven statistical threshold mechanism, as formal threshold testing and interaction analyses would be required to establish such a mechanism definitively. Notably, the relative yield increase with the B+M treatment under N reduction was consistent between the pot experiment and the field experiment. This parallel response suggests that the beneficial effects observed in pots may translate to field conditions, although the underlying mechanisms require direct investigation in future field-based microbial studies.

## 5 Conclusion

This study suggests a microbiome-mediated pathway through which combined B+M influence rice production in a N-dependent manner. B+M with 0% and 20% N reduction increased rice yield by 10.4-11.7% and 4.1-4.9%, whereas 40% reduction (N60) decreased yield by 3.9–4.6%, revealing a threshold-like pattern at 20% N reduction. SEM validated that soil bacterial communities drove yield enhancement (χ²/df = 1.13, CFI = 0.961, RMSEA = 0.042). Under N100 and N80, B+M enriched beneficial phyla (Proteobacteria, Actinobacteria) and upregulated metabolic pathways, improving soil nutrient availability. However, at 40% N reduction, key bacterial abundance declined and microbial functions weakened, reducing soil nutrient supply and yield. These findings identify 20% N reduction with B+M as a science-based strategy for sustainable rice production, balancing productivity and environmental safety, while highlighting the need for further mechanistic validation through targeted approaches such as gene expression analysis or isotope tracing.

## Data Availability

The raw sequencing data have been deposited in the NCBI Sequence Read Archive (SRA) under accession number PRJNA1434724.
