# Supplementary material for: Synergistic effects of biochar and microbial inoculants on rice productivity and soil fertility are mediated by a nitrogen-dependent microbial pathway
Source: Front Plant Sci. 2026 Apr 14;17:1804182. doi: 10.3389/fpls.2026.1804182 (PMC13123419; doi:10.3389/fpls.2026.1804182)
Supplement: Supplementary file 1 [file SupplementaryFile1.docx]

Supplementary Material

# Supplementary Figures and Tables

##
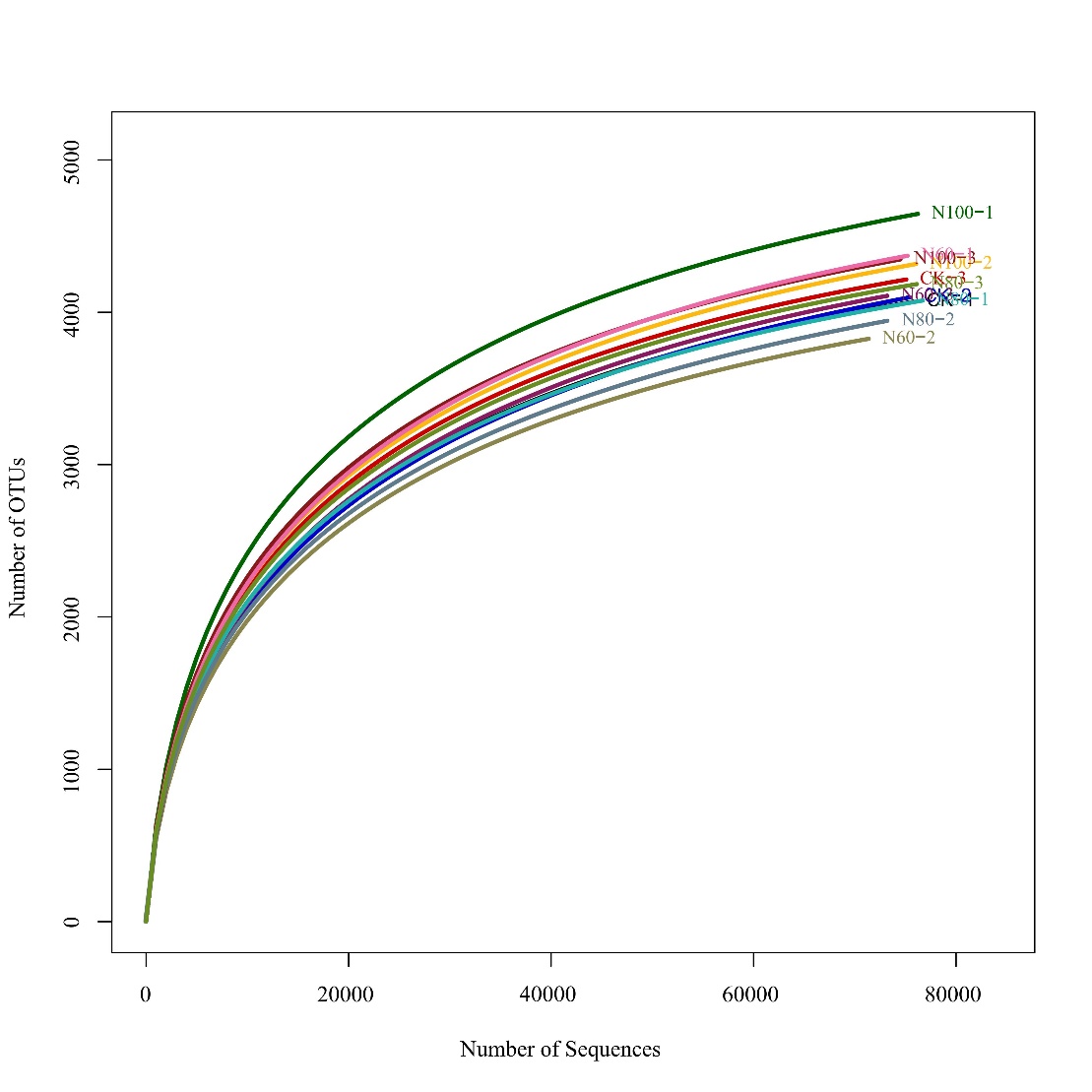
Supplementary Figures

**Figure S1.** Rarefaction curves of each sample at the 97% similarity level. Note: N100, N80, and N60 denote nitrogen reduction rates of 0%, 20%, and 40% under the combined application of biochar and microbial inoculants. CK represents conventional nitrogen rate with no soil amendments added.

| 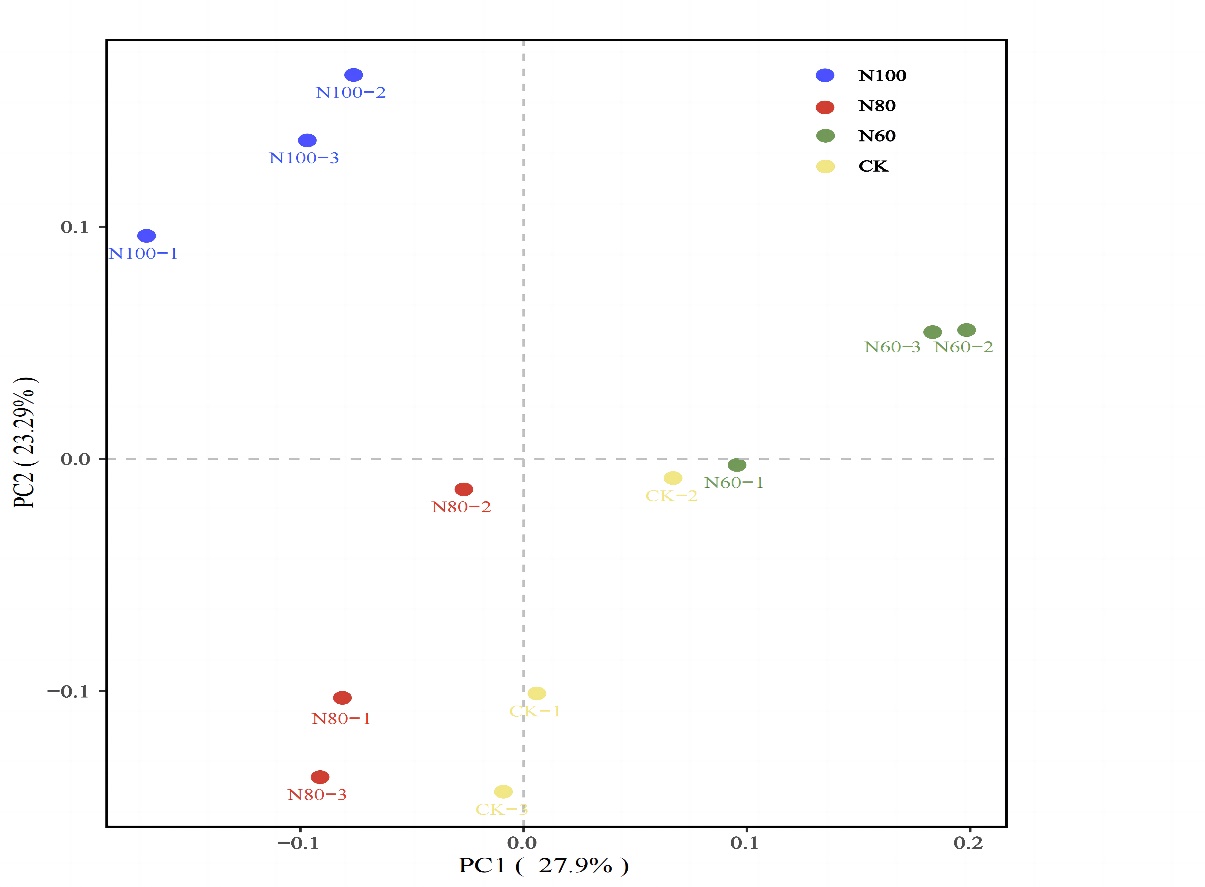 |
| --- |
| **Figure S2.** Effects of combined application of biochar and microbial inoculants on beta diversity of soil bacterial community in soil. Note: N100, N80, and N60 denote nitrogen reduction rates of 0%, 20%, and 40% under the combined application of biochar and microbial inoculants. CK represents conventional nitrogen rate with no soil amendments added. |

## Supplementary Tables

**Table S1.** N application rates for different treatments in pot experiment (g/pot).

| Treatment | Basal Fertilizer | Tillering Fertilizer | Panicle Fertilizer | Grain Fertilizer | Total Nitrogen |
| --- | --- | --- | --- | --- | --- |
|  | Urea | | | | |
| N100 | 2.05 | 2.05 | 1.37 | 1.37 | 6.85 |
| N80 | 1.64 | 1.64 | 1.10 | 1.10 | 5.48 |
| N60 | 1.23 | 1.23 | 0.82 | 0.82 | 4.11 |
| N0 | 0.00 | 0.00 | 0.00 | 0.00 | 0.00 |

**Table S2.** N application rates for different treatments in field experiment (kg ha^⁻1^).

| Treatment | Basal Fertilizer | Tillering Fertilizer | Panicle Fertilizer | Grain Fertilizer | Total Nitrogen |
| --- | --- | --- | --- | --- | --- |
| N100 | 81.0 | 81.0 | 54.0 | 54.0 | 270 |
| N80 | 64.8 | 64.8 | 43.2 | 43.2 | 216 |
| N60 | 48.6 | 48.6 | 32.4 | 32.4 | 162 |
| N0 | 0.0 | 0.0 | 0.0 | 0.0 | 0 |

**Table S3.**  Statistics of valid sequences.

| Sample | Sequences | Bases(bp) | Average Length(bp) |
| --- | --- | --- | --- |
| CK-1 | 95443 | 40023494 | 419.34 |
| CK-2 | 94270 | 39509590 | 419.11 |
| CK-3 | 94373 | 39578941 | 419.39 |
| N100-1 | 96133 | 40246710 | 418.66 |
| N100-2 | 97263 | 40754555 | 419.01 |
| N100-3 | 93366 | 39127696 | 419.08 |
| N60-1 | 96478 | 40455023 | 419.32 |
| N60-2 | 92421 | 38826177 | 420.10 |
| N60-3 | 93264 | 39162307 | 419.91 |
| N80-1 | 95390 | 39985169 | 419.18 |
| N80-2 | 91643 | 38444596 | 419.50 |
| N80-3 | 95408 | 40003028 | 419.28 |

Note: Sample represents the sample name; Sequences indicates the number of optimized sequences used for sample analysis; Bases refers to the total base count of the optimized sequences; AverageLength denotes the average length of the optimized sequences.
